# Pancreatic colonization of fungi in the development of severe acute pancreatitis

**DOI:** 10.3389/fcimb.2022.940532

**Published:** 2022-07-29

**Authors:** Yasuo Otsuka, Ken Kamata, Kosuke Minaga, Tomohiro Watanabe, Masatoshi Kudo

**Affiliations:** Department of Gastroenterology and Hepatology, Kindai University Faculty of Medicine, Osaka-Sayama, Osaka, Japan

**Keywords:** acute pancreatitis, fungi, intestinal barrier, walled-off necrosis, cytokines

## Abstract

Acute pancreatitis is a common emergent disorder, a significant population of which develops the life-threatening condition, called severe acute pancreatitis (SAP). It is generally accepted that bacterial infection is associated with the development and persistence of SAP. In addition to bacterial infection, recent clinical studies disclosed a high incidence of fungal infection in patients with SAP. Moreover, SAP patients with fungal infection exhibit a higher mortality rate than those without infection. Although these clinical studies support pathogenic roles played by fungal infection in SAP, beneficial effects of prophylactic anti-fungal therapy on SAP have not been proved. Here we summarize recent clinical findings as to the relationship between fungal infection and the development of SAP. In addition, we discuss molecular mechanisms accounting for the development of SAP in the presence of fungal infection.

## Introduction

Acute pancreatitis (AP) is a sudden-onset gastrointestinal disorder, which usually requires hospitalization. Although most cases with AP are self-limiting, a significant fraction of patients with AP develops a life-threatening condition, called severe acute pancreatitis (SAP). Indeed, the mortality rate of SAP is estimated to be approximately 20% (Boxhoorn et al., 2020). Despite such a high mortality rate of SAP, no curative treatments have been established in AP since the pathogenesis of this emergent disorder has been poorly understood.

Over-activation of pancreatic digestive enzymes, especially trypsinogen, followed by autodigestion of the pancreatic tissues underlies the pathogenesis of AP (Watanabe et al., 2017). In the steady state, conversion of trypsinogen into trypsin occurs in the duodenum upon exposure to enterokinase (Logsdon and Ji, 2013). Excessive drinking of alcohol and/or intake of high fatty foods sometimes initiates ectopic and intrapancreatic activation of trypsinogen followed by autodigestion (Watanabe et al., 2017). This trypsin-centered view regarding the pathogenesis of AP has been supported by the fact that human hereditary pancreatitis arises from mutations of genes associated with trypsinogen activation (Whitcomb, 2010). It should be noted, however, that the pathogenesis of AP cannot be explained by the trypsin-centered view alone. Recent studies provide evidence that mice deficient in T7 trypsinogen, an isoform equivalent to cationic human trypsinogen, still get experimental acute and chronic pancreatitis (Dawra et al., 2011; Sah et al., 2013). These surprising experimental data strongly support the view that AP is driven by multiple factors and that ectopic activation of trypsinogen is one of pathogenic triggers for acute pancreatic injury.

It is well established that AP is characterized by intestinal barrier dysfunction caused by pro-inflammatory cytokine responses. Leaky gut syndrome induced by AP allows translocation of gut microorganisms into the pancreas and thereby promotes further pro-inflammatory cytokine responses (Watanabe et al., 2017). SAP leads to local and systemic complications such as pancreatic necrosis and sepsis (Frossard et al., 2008; Lankisch et al., 2015). These local and systemic complications have been considered to arise from invasion of the pancreatic tissue by gut bacteria and subsequent dissemination to systemic organs leading to endotoxemia. Furthermore, the presence of multiple organ failure and pancreatic necrosis has been identified as the prognostic factors for SAP as shown by the fact that the mortality rate of SAP is much higher in cases with infection than in those without bacterial infection (Petrov et al., 2010). In addition, recent experimental studies support the view that sensing of gut bacteria followed by pro-inflammatory cytokine responses play pathogenic roles in the development of SAP. Recognition of gut Gram-negative bacteria by toll-like receptor 4 (TLR4) and nucleotide-binding oligomerization domain 1 (NOD1) expressed by pancreatic acinar cells and immune cells causes pro-inflammatory cytokine responses in experimental pancreatitis (Sharif et al., 2009; Tsuji et al., 2012; Watanabe et al., 2016). Therefore, these clinical and experimental studies strongly suggest that pro-inflammatory cytokine responses caused by translocation of gut bacteria into the pancreas act in concert with intrapancreatic activation of trypsin to induce SAP. Thus, pancreatic infection with gut bacteria is one of the most pathogenic events in the development of SAP.

Given the fact that gut microorganism community is composed not only of bacteria but also of fungi, it is rational to assume that translocation and colonization of gut fungi into the pancreas is associated with the development of SAP. Indeed, gastroenterologists often encounter with SAP cases exhibiting candidemia (Rasch et al., 2018). Although pathogenic roles played by pancreatic infection with gut bacteria have been established, those played by pancreatic colonization of gut fungi have not been fully understood. In addition, it remains controversial whether pancreatic colonization of gut fungi is a bystander phenomenon or pathogenic event. In this Minireview article, we summarize recent findings as to the relationship between SAP and fungal infection.

## Link between bacterial infection and human SAP

AP is classified into interstitial edematous pancreatitis and necrotizing pancreatitis based on the characteristic findings of dynamic computed tomography (CT). Interstitial edematous pancreatitis can be diagnosed by the presence of pancreas swelling alone whereas appearance of hypo-enhancement areas and fluid collection in the pancreas itself or surrounding the pancreas in the dynamic CT study suggests necrotizing pancreatitis. Pancreatic necrosis develops early in the clinical course of SAP (Mederos et al., 2021). Necrotizing pancreatic tissue is further subclassified into acute necrotic collection (ANC) and walled-off necrosis (WON) by the duration displaying fluid collection and necrotic materials. Pancreatic and/or peripancreatic fluid collection and necrotic areas detected within four weeks from the onset are regarded as immature and non-sequestrated forms of pancreatic necrosis and thus called as ANC. Pancreatic necrosis, which persists more than four weeks after the onset, leads to the generation of matured and encapsulated forms of necrotic tissues, called WON (Banks et al., 2013). Although both ANC and WON are composed of fluid and necrotic materials, the latter form, but not the former form, is a matured and encapsulated form of pancreatic necrosis. Discrimination of WON from ANC is very important since necrosectomy is usually required for the treatment of WON (van Santvoort et al., 2010).

Colonization of gut bacteria into the pancreas plays critical roles in the development of ANC or WON and progression of ANC into WON (Dragonetti et al., 1996; van Minnen et al., 2006). In fact, Gram-negative rods in the intestine are the most frequently isolated and identified species from local or blood cultures of pancreatic infections, with *E. coli* being the most frequently reported (Ignatavicius et al., 2012). Moreover, ANC or WON has been implicated as a reservoir for systemic dissemination of gut bacteria (Bradley et al., 2008). Thus, it is no doubt that translocation of gut bacteria into the pancreas followed by colonization and infection is an indispensable step for the development of SAP displaying ANC or WON. Strong support for this idea comes from a meta-analysis using six randomized controlled trials showing that prophylactic antimicrobial therapy reduced mortality and complication rates associated with pancreatic infection (Ukai et al., 2015).

## Link between fungal infection and human SAP

Increased intestinal permeability associated with SAP may permit entry not only of bacteria but also of fungi into the pancreas. Therefore, it is likely that translocation of gut fungi into the pancreas promotes the development of SAP displaying ANC or WON. Indeed, there are several reports showing the association between SAP and fungal infection (Kochhar et al., 2011; Rasch et al., 2018; Singh et al., 2021). As shown in **Table 1**, the rates of fungal infection as assessed by isolation of blood or pancreatic tissues or by the presence of β-d-glucan antigenemia are highly variable ranging from 7.6% to 46.3% in patients with AP (Kochhar et al., 2009; Vege et al., 2009; Ignatavicius et al., 2012; Werge et al., 2016; Mandal et al., 2017; Rasch et al., 2018; Ning et al., 2021). Such highly variable results can be explained by the difference of severity in patients with AP; the incidence of pancreatic fungal infection is parallel to the severity of AP. In fact, pancreatic infection with fungi occurs in around 40% of SAP patients bearing WON (Werge et al., 2016; Rasch et al., 2018). Rasch et al. examined the incidence of *Candida* infection in patients with pancreatic necrosis and found that *Candida* species were isolated from the pancreatic necrotic tissues in 54 patients among total 136 patients with pancreatic necrosis (Rasch et al., 2018). More importantly, patients positive for pancreatic *Candida* infection exhibited a higher mortality rate as compared with those negative for *Candida* infection (35.2% vs. 13.4%) (Rasch et al., 2018). Such a high mortality rate observed in patients with pancreatic *Candida* infection might be partially explained by the presence of candidemia since the highest mortality rate was observed in patients with both candidemia and pancreatic *Candida* infection (Rasch et al., 2018). Thus, disseminated *Candida* followed by pancreatic infection with this organism or vice versa determines the prognosis of patients with SAP. Consistent with these data, the latest meta-analysis, in which the incidence of *Candida* infection and its impact on mortality were studied in 22 reports, also verified that pancreatic or systemic *Candida* infection increases the mortality (Singh et al., 2021).

**Table 1:** Fungal infection in severe acute pancreatitis.

Author	Patients, n	Fungal infection cases, n (%)	WON cases, n (%)	Antifungal therapy		Mortality, n (%)
				Prophylactic, n (%)	Symptomatic, n (%)	
Ning et al (2021)	78	8 (10.2)	NA	0 (0)	8 (10.2)	NA
Rasch et al (2018)	136	54 (39.7)	136 (100)	0 (0)	36 (26.4)	30 (22.0)
Mandal et al (2017)	76	NA	9 (6.6)	NA	NA	1 (1.3)
Werge et al (2016)	123	57 (46.3)	123 (100)	0 (0)	39 (31.7)	22 (17.8)
Ignatavicius et al (2012)	210	16 (7.6)	31 (14.8)	NA	NA	34 (16.2)
Vege et al (2009)	207	30 (14.4)	107 (51.6)	19 (9.1)	NA	41 (19.8)
Kochhar et al (2009)	50	18 (36.0)	25 (50.0)	0 (0)	13 (26.0)	21 (42.0)

NA, not applicable; WON, walled-off necrosis.

In another report, fungal infection has been shown to be associated with the development of WON (Werge et al., 2016). Werge et al. retrospectively analyzed the incidence of fungal infection in SAP patients bearing WON and found that fungi were isolated in necrotic pancreatic tissues or fluids in 57 patients among total 123 patients (Werge et al., 2016). Such high incidence of fungal infection in SAP patients bearing WON strongly suggests involvement of fungal infection in the maturation and encapsulated process of pancreatic necrosis. Confirmation of this idea requires studies directly comparing the incidence of pancreatic fungal infection in SAP patients with ANC or WON. However, the incidence of pancreatic fungal infection in SAP patients bearing ANC has not been examined since necrosectomy is not usually performed and thus pancreatic necrotic tissues are not obtained for the isolation of fungi in such cases. Another important issue that need to be addressed is which is more critical for the prognosis of SAP, bacterial or fungal infection. In this regard, SAP patients with intraabdominal fungal infection exhibited longer hospital and intensive care unit stays than those with bacterial infection (Werge et al., 2016).

*Candida* species have been identified as the most common organism associated with SAP (Grewe et al., 1999). Chakrabarti et al. reported that *Candida tropicalis* (43.9%) and *Candida albicans* (36.6%) were the most common isolates from pancreatic tissues of 335 SAP patients with fungal infection (Chakrabarti et al., 2007). Similarly, *Candida albicans* (55%) and *Candida glabrada* (20%) are frequently isolated from WON (Werge et al., 2016). On the other hand, *Candida krusei* (4.5%) is a minor population of pancreatic fungal community in many cases with pancreatic necrosis (Chakrabarti et al., 2007). There are also reports suggesting involvement of infection with *Torulopsis glabrata* or *Saccharomyces* (Grewe et al., 1999; Berzin et al., 2007). In contrast, infection with *Aspergillus* species, prototypical commensal fungi in the respiratory tract, has been rarely reported except in immunosuppressed SAP patients (Bhatt and Cappell, 1990). Thus, *Candida* species have been identified as virulence fungi in the development of SAP.

High detection rates of *Candida* species from the blood and pancreatic tissues in patients with SAP implicate possible translocation pathways of this microorganism (Rasch et al., 2018). Given that *Candida* species is predominant fungi in commensal fungal community of human gastrointestinal (GI) tract (Pérez, 2021), *Candida* species are likely to migrate from the gut to the pancreas upon intestinal barrier injury associated with SAP. However, we need to consider translocation of *Candida* species from the skin to the pancreas since *Candida* species constitute skin fungal community (Brown et al., 2012). It should be noted, however, that previous studies have utilized conventional culture methods for isolation of fungi. Therefore, a comprehensive evaluation of the fungal flora in pancreatic tissues and blood has not been achieved, and it is unknown which types of fungal flora dysbiosis may affect the development of SAP. Next generation sequence (NGS) analyses targeting fungal internal transcribed spacer region of the nuclear ribosomal repeat have been employed to visualize composition of fungal species (Toju et al., 2012). Given that isolation of fungal species was performed by conventional culture methods alone, application of NGS utilizing necrotic pancreatic specimen and/or blood may enable us to understand the fungal community and diversity associated with the development of WON.

## Anti-fungal therapy in SAP

It is generally accepted that anti-microbial therapy needs to be started for patients with AP when the infection with bacteria or fungi is highly suspected by the serum detection of microbial components such as endotoxin or β-d-glucan or by the presence of bacteremia or fungemia (King et al., 2005; Kochhar et al., 2009; Vege et al., 2009). In cases with fungal infection, administration of amphotericin B and/or fluconazole is required (King et al., 2005; Kochhar et al., 2009; Vege et al., 2009). Consistent with this idea, prophylactic and early administration of fluconazole significantly reduced the incidence of fungal infection in patients with SAP (De Waele et al., 2003; He et al., 2003). However, prophylactic fluconazole administration failed to improve survival despite a significant reduction in fungal infection (Shorr et al., 2005). Furthermore, another report identified prophylactic fluconazole administration as one of the risk factors for *Candida* infection in the pancreatic necrotic tissues (Chakrabarti et al., 2007). Thus, beneficial effects of prophylactic anti-fungal therapy on SAP have not been demonstrated and prophylactic anti-fungal therapy is not recommended according to current guidelines for AP (Tenner et al., 2013; Crockett et al., 2018; Leppäniemi et al., 2019; Takada et al. 2022). Several studies addressed the efficacy of anti-fungal therapy in relation to the incidence of WON (**Table 1**). As mentioned above, fungal infection rates in the patients with acute pancreatitis ranged from 7.6 to 46.3%. Notably, fungal infection rates tended to be higher in the patients with WON than in the patients without WON, suggesting pathogenic roles played by fungal infection in the development of WON.

As for the efficacy of anti-fungal therapy consisting of amphotericin B and/or fluconazole for SAP, four studies started anti-fungal therapy after isolation of fungi (Kochhar et al., 2009; Werge et al., 2016; Rasch et al., 2018; Ning et al., 2021). On the other hand, antifungal therapy was initiated prophylactically in one study in which prophylactic antifungal therapy was performed in 19 cases due to β-D-glucan positivity in the serum (Vege et al., 2009) despite without fungal detection in culture tests. As shown in **Table 1**, the mortality rates are comparable whether anti-fungal therapy is initiated after the isolation of fungi or detection of β-D-glucan. In terms of the mortality rates, administration of anti-fungal agents upon detection of serum β-D-glucan might not be beneficial for patients with SAP. However, Tissot et al. reported superiority of β-D-glucan detection for the diagnosis of intraabdominal *Candida* infection as compared with conventional culture methods (Tissot et al., 2013). Alternatively, the presence of multiple organ failure might be an indication for prophylactic anti-fungal therapy (Ning et al., 2021). Further studies addressing the efficacy and timing of anti-fungal therapy for SAP are absolutely required.

Anti-fungal therapy is sometimes accompanied by side effects such as liver dysfunction, kidney dysfunction, and allergy (Nivoix et al., 2020). We have to be cautious regarding the doses of amphotericin B and/or fluconazole since patients with SAP usually have liver and kidney dysfunction as a result of multiple organ failure. In addition, another concern is an emergence of multidrug-resistant organisms due to the long-term administration of antifungal drugs. Indeed, cases with drug-resistant *Candida glabrata* infection are increasing after anti-fungal therapy (Pfaller et al., 2012).

Duration of antibiotics has been shown to increase the risk of fungal infection in patients with SAP (Kochhar et al., 2011). As in the case of fungal infection, current guidelines do not recommend prophylactic antibiotics therapy for all of the cases with AP (Tenner et al., 2013; Crockett et al., 2018; Leppäniemi et al., 2019; Takada et al., 2022). It is because meta-analysis using seven randomized controlled trials showed no significant improvement with prophylactic anti-microbial therapy in both mortality or infectious complication rates in SAP (Tenner et al., 2013; Crockett et al., 2018; Leppäniemi et al., 2019; Takada et al., 2022). Thus, no consensus has been reached regarding the timing of anti-bacterial or anti-fungal therapy.

Involvement of pathogenic immune responses against fungi has been extensively studied in patients with inflammatory bowel disease (IBD) and thus we can learn management of fungal infection from clinical practice in IBD (Underhill and Braun, 2022). A significant fraction of IBD patients have elevated levels in serum antibodies against *Saccharomyces cerevisiae* and polymorphisms in *CARD9* (*Caspase activation and recruitment domain 9*) encoding CARD9, a critical intracellular molecule for immune responses against fungi, are associated with the development of IBD (Underhill and Braun, 2022). Furthermore, clinical responses in fecal microbiota transplantation (FMT) in ulcerative colitis are associated with reduction of *Candida* species after FMT (Leonardi et al., 2020). Despite these extensive studies in fungal communities in IBD, no definitive anti-fungal therapy has been established. Thus, further basic and clinical studies are required to clarify the molecular mechanisms accounting for the link between fungi infection and IBD or SAP and then to establish anti-fungal therapy for these disorders.

## Molecular mechanisms accounting for the development of SAP by colonization of fungi into the pancreas

As mentioned above, clinical studies clearly showed that the incidence of fungal infection is high in SAP patients bearing WON and that fungal infection is one of the prognostic factors. However, beneficial effects of prophylactic anti-fungal therapy have not been demonstrated so far. Elucidation of molecular mechanisms how pancreatic colonization of fungi leads to the development of SAP displaying WON is required to establish new treatment strategies against fungal infection associated with SAP.

**Figure 1:**
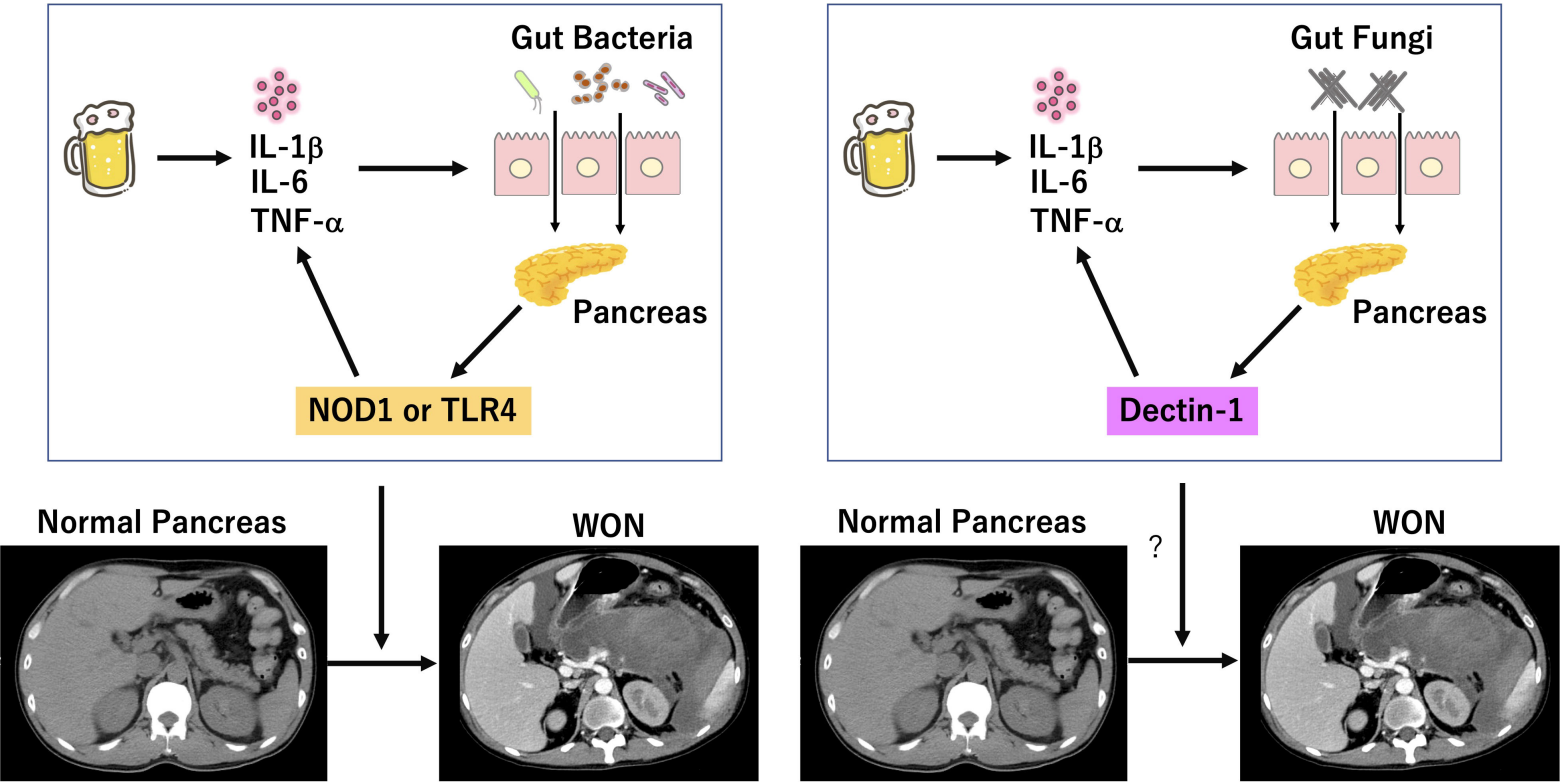
Involvement of bacterial and fungal infection in the development of severe acute pancreatitis. Excessive drinking of alcohol induces auto-digestion of pancreatic tissues due to intrapancreatic activation of trypsinogen. Pro-inflammatory cytokine responses induced by initial inflammation dampen intestinal barrier function and allow entry of gut bacteria into the pancreas. Sensing of bacteria by nucleotide-binding oligomerization domain 1 (NOD1) and toll-like receptor 4 (TLR4) leads to a robust production of pro-inflammatory cytokines, which in turn increases intestinal permeability and translocation of gut bacteria. Intestinal barrier dysfunction also allows entry of gut fungi into the pancreas. Sensing of fungi by Dectin-1 may lead to a robust production of pro-inflammatory cytokines, which in turn increases intestinal permeability and translocation of gut fungi. Positive feedback loop between intestinal gut barrier dysfunction and pro-inflammatory cytokine responses induced by colonization of bacteria and/or fungi may be involved in the development of SAP displaying walled-off necrosis (WON). We created the Figure, which consisted of original cartoon pictures and CT pictures from patients who visited our hospital. CT pictures have not been published. This figure is original and based on our proposal and assumption.

SAP is characterized by impaired intestinal barrier function and pro-inflammatory cytokine responses (Wu et al., 2014; Watanabe et al., 2017). Potential triggers such as excessive drinking of alcohol cause pro-inflammatory cytokine responses due to intrapancreatic activation of trypsin and subsequent auto-digestion of the pancreatic tissues (Watanabe et al., 2017). Pro-inflammatory cytokines such as TNF-α, IL-1β, and IL-6 increase the intestinal permeability and then as a result translocation of gut bacteria into the pancreas is promoted (Watanabe et al., 2017). Pancreatic acinar cells, macrophages, and dendritic cells express functional pattern recognition receptors (PRRs) for the detection of bacterial components. NOD1 and TLR4 are the prototypical PRRs for the detection of cell wall components derived from gut bacteria translocated into the pancreas. Recognition of bacterial cell wall components by NOD1 and TLR4 leads to a robust production of pro-inflammatory cytokines by immune cells and acinar cells, which in turn further increases the intestinal permeability and accelerates translocation of gut bacteria. Such positive feedback loop connecting pro-inflammatory cytokines and impaired intestinal barrier function have been considered to underlie the immunopathogenesis of SAP and to contribute to the development of SAP displaying WON (**Figure 1**) (Watanabe et al., 2017). Based on the inflammatory cascades leading to the development of SAP, one might assume that SAP can be efficiently treated by the neutralization of PRRs-mediated signaling pathways (Watanabe et al., 2017). In fact, mice deficient in TLR4 or NOD1 are highly resistant to induction of experimental pancreatitis (Sharif et al., 2009; Tsuji et al., 2012; Watanabe et al., 2016). Alternatively, SAP can be treated by biologics targetting TNF-α, IL-6, or IL-1β as in the case of autoimmune disorders (Watanabe et al., 2017). Previous trials addressing the efficacy of prophylactic antibiotic treatment for the prevention of SAP were not successful (Takada et al., 2022). Although the reasons for the unsuccessful results remain unknown, overgrowth of antibiotic-resistant bacteria and fungi may be involved. Therefore, inflammatory cascades linking bacterial colonization to pro-inflammatory cytokines can be new treatment targets for SAP.

C-type lectin receptors (CLRs) are the main family of PRRs for the detection of fungal cell wall components (Netea et al., 2015). β-d-glucan antigenemia is widely used for the early detection of candidemia in patients with SAP. β-d-glucans derived from *Candida* cell walls are strong stimulators for Dectin-1 expressed in macrophages and dendritic cells (Netea et al., 2015). Recognition of β-d-glucans by Dectin-1, a prototypical CLRs, leads to an increased production of pro-inflammatory cytokines through activation of downstream signaling molecule, spleen tyrosine kinase (SYK). Therefore, the presence of β-d-glucan antigenemia in patients with SAP may mean enhancement of Dectin-1-mediated pro-inflammatory cytokine responses, which result in translocation of gut fungi into the pancreas due to leakiness of the barrier (**Figure 1**). Based on this, we propose the following mechanism as to involvement of fungal infection in the development of SAP. Initial pro-inflammatory cytokine responses caused by intrapancreatic activation of trypsinogen and subsequent auto-digestion impairs intestinal barrier function, which allows pancreatic entry of gut fungi. Migrated fungi are recognized by Dectin-1 expressed in pancreatic macrophages and dendritic cells to induce pro-inflammatory cytokine responses and to promote maturation of pancreatic necrotic tissues leading to generation of WON. Thus, we assume that inflammatory cascades linking pancreatic fungal colonization to pro-inflammatory cytokines mediates the development of SAP as in the case of pancreatic bacterial colonization. This scenario explains the fact fungal infection is preferentially detected in patients with SAP displaying WON. Although no studies have addressed this issue in experimental models of AP, fungi colonized in the pancreas have been shown to promote inflammatory as well as oncogenic pathways in pancreatic cancer (Aykut et al., 2019; Alam et al., 2022).

## Conclusions

Colonization of fungi into the pancreas is associated with the development of SAP displaying WON and high mortality rates although beneficial effects of prophylactic anti-fungal therapy on SAP have not been confirmed. Elucidation of molecular mechanisms accounting for the relationship between WON and fungi infection is required to establish treatment strategies against fungal infection in AP. As far as we know, few studies addressed involvement of fungi in experimental models of SAP. Moreover, previous studies as to the identification of fungal species utilized conventional culture methods, but not NGS. Comprehensive understanding of involvement of fungi in SAP requires both animal and human studies employing NGS to uncover overlooked fungi species. For this purpose, it is necessary to examine whether fungal dysbiosis is associated with the development of animal and human SAP through visualization of gut and pancreatic fungal community by NGS.

## Author contributions

YO, KK, and TW wrote the manuscript draft. KK, TW, KM, and MK revised the manuscript. All authors contributed to the article and approved the submitted version.

## Funding

This work was supported by Grants-in-Aid for Scientific Research (21K159857, 22K07996, 22K08090) from the Japan Society for the Promotion of Science, Takeda Science Foundation, Smoking Research Foundation, Yakult Bio-Science Foundation, SENSHIN Medical Research Foundation, and 2022 Kindai University Research Enhancement Grant (KD2208).

## Conflict of interest

The authors declare that the research was conducted in the absence of any commercial or financial relationships that could be construed as a potential conflict of interest.

## Publisher’s note

All claims expressed in this article are solely those of the authors and do not necessarily represent those of their affiliated organizations, or those of the publisher, the editors and the reviewers. Any product that may be evaluated in this article, or claim that may be made by its manufacturer, is not guaranteed or endorsed by the publisher.
